# Network meta-analysis of migraine disorder treatment by NSAIDs and triptans

**DOI:** 10.1186/s10194-016-0703-0

**Published:** 2016-12-12

**Authors:** Haiyang Xu, Wei Han, Jinghua Wang, Mingxian Li

**Affiliations:** The First hospital of Jilin University, No. 71 Xinmin Street, Changchun, 130021 Jilin China

**Keywords:** Migraine disorders, Triptans, Non-steroidal anti-inflammatory agents, Network meta-analysis

## Abstract

**Background:**

Migraine is a neurological disorder resulting in large socioeconomic burden. This network meta-analysis (NMA) is designed to compare the relative efficacy and tolerability of non-steroidal anti-inflammatory agents (NSAIDs) and triptans.

**Methods:**

We conducted systematic searches in database PubMed and Embase. Treatment effectiveness was compared by synthesizing direct and indirect evidences using NMA. The surface under curve ranking area (SUCRA) was created to rank those interventions.

**Results:**

Eletriptan and rizatriptan are superior to sumatriptan, zolmitriptan, almotriptan, ibuprofen and aspirin with respect to pain-relief. When analyzing 2 h-nausea-absence, rizatriptan has a better efficacy than sumatriptan, while other treatments indicate no distinctive difference compared with placebo. Furthermore, sumatriptan demonstrates a higher incidence of all-adverse-event compared with diclofenac-potassium, ibuprofen and almotriptan.

**Conclusion:**

This study suggests that eletriptan may be the most suitable therapy for migraine from a comprehensive point of view. In the meantime ibuprofen may also be a good choice for its excellent tolerability. Multi-component medication also attracts attention and may be a promising avenue for the next generation of migraine treatment.

**Electronic supplementary material:**

The online version of this article (doi:10.1186/s10194-016-0703-0) contains supplementary material, which is available to authorized users.

## Background

Migraine is a neurological disorder resulting in large socioeconomic burden affecting approximately 18% of females and 6% of males in the United States [1]. The prevalence of migraine varies with age, females between 35 and 45 years old exhibits the highest prevalence [2]. Apart from the factor of age, the prevalence of migraine in the U.S. also varied with household income and race, and such findings are consistent with studies carried out in other countries [3, 4]. Headache is the primary symptom of migraine and patients may also be afflicted by other symptoms including pulsatile pain, light sensitivity, sound sensitivity, nausea, unilateral pain, blurred vision and emesis. Although a large number of treatments have been developed for migraine over the past decades, several disputes have been encountered by clinicians such as misclassification of migraine, inappropriate selection of treatment and medication overuse. Among them, medication overuse has become a major issue in chronic migraine patients who may eventually develop a disabling condition called medication-overuse headache [5]. Therefore, awareness and understanding of migraine should be improved and corresponding treatments or medications should be further explored to overcome these issues.

Two types of migraine therapies have been developed: preventive therapies which are used to reduce attack frequency or severity and acute therapies which are used for the sake of aborting attacks. Compared to preventive therapies, acute therapies are able to provide patients with rapid and complete relief with minimal or no adverse events and hence they are recommended for promptly alleviating the symptoms of patients [6]. The selection of acute treatments has been differentiated into two pathways: non-specific medications which include analgesics and non-steroidal anti-inflammatory drugs (NSAIDs); and specific medications which include ergot derivatives and triptans [5]. As suggested by the European Federation of Neurological Societies (EFNS), both oral NSAIDs and triptans are recommended for treating migraine attacks [7]. Moreover, evidence from the American Headache Society (AHS) concluded that the following treatments are deemed to be effective acute therapies for migraine: triptans, NSAIDs, ergotamine derivatives, opioids and other combinational medications [8]. Stratified care is a primary strategy often used in selecting medications for migraine patients and this strategy takes several aspects into account: attack severity, the presence of associated symptoms and the degree of disability resulting from migraine [9]. However, other factors such as dosage may also have significant influence on the overall effectiveness of medications that are used to abort migraine attacks.

Among the common acute treatments that are used for aborting migraine, different levels of evidence have been provided by a wide range of studies. Although the efficacy of some medications have been established, this does not imply that such medications should be considered as the first line treatments for migraine patients since it may cause adverse events that are specifically associated with these medications. Despite the growing popularity of triptans, NSAIDs remain one of the most recommended acute migraine treatments and they are often used as an initial strategy for aborting migraine attacks [9]. On the other hand, triptans are often used as a rescue medication if an initial treatment fails to abort migraine attacks and evidence suggests that about 60% of non-responders to NSAIDs can be treated by triptans [10]. One distinctive advantage of triptans for migraine patients is that they can be effective at any time during a migraine attack and such an advantage may reduce the impact of dosage timing on the overall efficacy. Moreover, some evidence suggests that earlier intervention by using triptans is associated with an enhanced efficacy [11, 12], while some randomized trials do not support such an improved efficacy when patients experienced allodynia in the course of a migraine attack [13, 14].

Despite the fact that both NSAIDs and triptans have been recommended by the EFNS and AHS as acute treatments for migraine, comparing NSAIDs with triptans is a challenging task. Conventional meta-analysis has several limitations due to the lack of evidence as well as lack of indirect evidence.. For this reason, we designed this network meta-analysis (NMA) to compares the relative efficacy and tolerability between NSAIDs and triptans. We hope that the approach of NMA can provide comprehensive evidence with respect to the efficacy and tolerability of these two popular medications.

## Methods

### Search strategy

We employed search strategies to explore the medical literature for relevant studies in PubMed and EMBASE systematically, and 2,967 records were identified using the following terms: “migraine disorders”, “tryptans”, “non-steroidal anti-inflammatory agents”, “ergot alkaloids”, “opioid analgesics”, “sumatriptan”, “zolmitriptan”, “almotriptan”, “rizatriptan”, “naratriptan”, “ibuprofen”, “eletriptan”, “diclofenac-potassium” and “aspirin” in PubMed. Reviewers also provided 3 additional references.

**Fig. 1:**
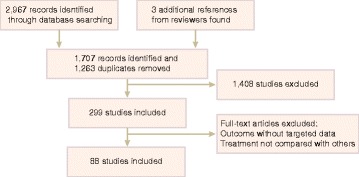
Study flow diagram

As flow chart Fig. 1 illustrates, among the total 2,970 records, 1,263 were identified as duplicates and hence removed after assessment. 1,408 more studies were excluded from the remaining 1,707 records according to the exclusion criteria, leaving 299 remnant studies. Full-text articles were viewed and included if they met the inclusion criteria, or excluded if not. Eventually 88 studies were included in this research [12, 15–101].

### Inclusion criteria

Articles were included if they: (1) were randomized clinical trials (RCTs); (2) were categorized as double blind; (3) included relevant clinical outcomes and treatments; (4) contained comparisons between different treatments.

### Outcome measures and data extraction

**Fig. 2:**
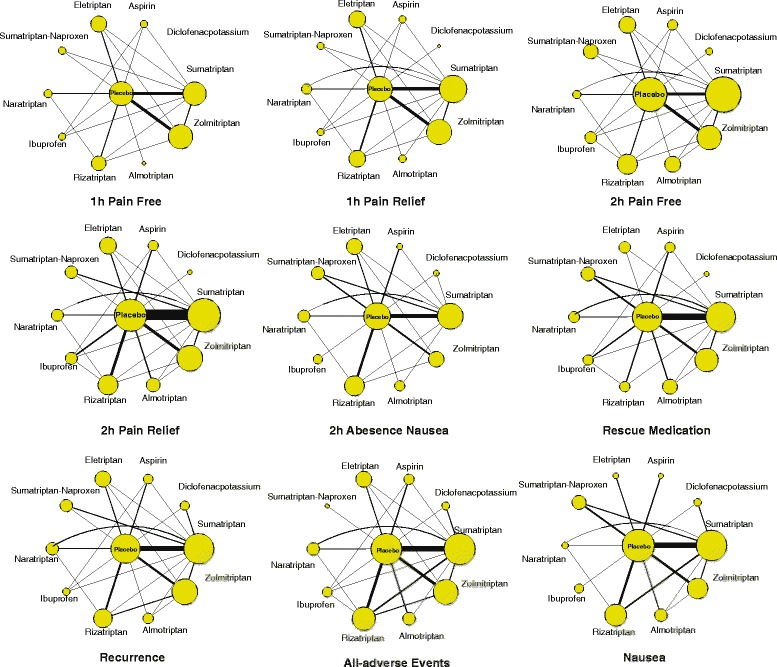
Network of randomized controlled trials comparing different medications agents of migraine treatments. The width of the lines is proportional to the number of trials comparing each pair of treatments; the area of circles represents the cumulative number of patients for each intervention

The following data were extracted from eligible studies and shown in Table 1: gender, sample size and diagnostic criteria. Two investigators reviewed the manuscripts of all included studies and extracted data into a database independently. A Jadad scale was generated and is presented in Additional file 1: Table S1. The width of the lines in Fig. 2 is proportional to the number of trials comparing each pair of treatments and the area of circles represents the cumulative number of patients for each intervention.

**Table 1:** Included studies

Study information	Blinding	Number	Female	Diagnostic criteria	Outcomes
Sumatriptan vs Placebo					
Barbanti, 2004, Multinational	Double	432	358	IHS	③
Bigal, 2015, USA	Double	354	386	ICHD-II	③④
Bousser, 1993, France	Double	96	79	IHS	③④⑤⑥⑧
Cady, 1998, USA	Double	132	112	IHS	④⑥⑦⑧
Cady, 2015, USA	Double	212	177	ICHD-II	③④⑤⑥
Diamond, 1998, USA	Double	1077	956	IHS	②④⑤
Diener, 1999, Germany	Double	156	125	IHS	③④⑥⑦⑧⑨
Djupesland, 2010, UK	Double	78	71	IHS	②③④⑥⑧
Fujita, 2014, Japan	Double	144	84	ICHD-II	①②③④⑧⑨
Goldstein, 2005, USA	Double	104	-	IHS	⑥⑦
Gross, 1994, UK	Double	86	69	IHS	②⑥
Henry, 1993, France	Double	76	66	IHS	①②③④⑥⑧
Jelinski, 2006, USA	Double	235	308	IHS	①③⑨
Landy, 2004, UK	Double	449	448	IHS	③⑨
Lipton, 2000, USA	Double	1112	215	IHS	③④
Myllyla, 1998, Finland	Double	94	84	IHS	③④⑤⑥⑦
Nappi, 1994, Italy	Double	244	155	IHS	③④⑤⑥⑦⑧⑨
Peikert, 1999, Multinational	Double	586	408	IHS	②③④⑧
Pini, 1995, Italy	Double	240	-	IHS	④⑥⑦⑧
Rao, 2016, USA	Double	100	54	IHS	③④⑤⑨
Salonen, 1994, Multinational	Double	247	30	IHS	②④
Schulman, 2000, USA	Double	116	105	IHS	⑥⑦⑧
Sheftell, 2005, USA	Double	904	1170	IHS	③⑥⑦⑨
Tfelt-Hansen, 1995, Multinational	Double	248	192	IHS	④⑤⑥⑧⑨
Tfelt-Hansen, 2006, Denmark	Double	100	78	-	③⑧
Wang, 2007, Taipei	Double	56	48	-	①②③④⑦⑧⑨
Wendt, 2006, USA	Double	577	500	-	①②③④⑥⑧⑨
Winner, 2003, USA	Double	354	311	-	①③⑨
Winner, 2006, USA	Double	297	246	-	③④⑥⑨
Zolmitriptan vs Placebo					
Charlesworth, 2003, UK	Double	1372	1138	IHS	⑥⑦⑧
Dahlof, 1998, Multinational	Double	840	701	IHS	③④⑤⑥⑦⑧
Dodick, 2005, USA	Double	1868	1620	IHS	①②③④⑤⑥⑦⑨
Dowson, 2002, Multinational	Double	470	409	IHS	④⑥⑦⑧
Gawel, 2005, Canada	Double	912	798	IHS	①②③④⑥⑧⑨
Klapper, 2004, UK	Double	280	241	IHS	③⑥⑧⑨
Loder, 2005, USA	Double	565	482	IHS	①③⑧⑨
Rothner, 2006, USA	Double	346	410	IHS	①②③④⑧⑨
Ryan Jr, 2000, North America	Double	734	628	IHS	①②③④⑥⑦
Sakai, 2002, Japan	Double	202	150	IHS	②③④⑤⑧
Spierings, 2004, USA	Double	670	580	IHS	①②③④⑤⑧⑨
Tepper, 1999, Multinational	Double	1643	1387	IHS	③⑧
Tuchman, 2006, USA	Double	336	-	-	
Almotriptan vs Placebo					
Diener, 2005, Germany	Double	221	192	IHS	③④⑧
Dowson, 2002, Multinational	Double	470	409	IHS	④⑥⑦⑧
Mathew, 2007, USA	Double	317	275	IHS	①②③④⑧⑨
Pascual, 2000, Multinational	Double	909	788	IHS	①②③④⑤⑦⑧
Rizatriptan vs Placebo					
Ahrens, 1999, USA	Double	555	391	IHS	②③④⑤⑦⑨
Freitag, 2008, USA	Double	82	72	IHS	③④⑤⑨
Mannix, 2007, USA	Double	359	355	IHS	④⑤⑥⑧
Teall, 1998, Multinational	Double	762	653	IHS	②④⑦⑧⑨
Misra, 2007, India	Double	103	76	IHS	③④⑥⑧
Ibuprofen vs Placebo					
Codispoti, 2001, USA	Double	660	556	IHS	③④⑤⑧⑨
Goldstein, 2006, USA	Double	886	722	IHS	④⑨
Kellstein, 2000, USA	Double	729	550	IHS	③④⑤⑥
Misra, 2004, India	Double	105	57	-	④
Sumatriptan-Naproxen vs Placebo					
Mannix, 2009, USA	Double	314	313	-	③⑥⑨
Martin, 2014, USA	Double	623	622	ICHD-II	
Silberstein, 2014, USA	Double	443	331	ICHD-II	①②③⑤⑥⑧⑨
Winner, 2015, USA	Double	349	66	ICHD	③
Eletriptan vs Placebo					
Diener, 2002, Multinational	Double	530	465	IHS	②③④⑨
Diclofenacpotassium vs Placebo					
Comoglu, 2011, Turkey	Double	45	10	IHS	②
Diener, 2006, Germany	Double	590	762	IHS	③④⑥⑦⑨
Lipton, 2010, USA	Double	690	585	IHS	③⑦⑧
Aspirin vs Placebo					
Lange, 2000, Germany	Double	345	-	IHS	③④⑤⑥⑧
Lipton, 2005, USA	Double	401	317	IHS	③④⑥⑦⑧⑨
Sumatriptan vs Zolmitriptan					
Gallagher, 2000, USA	Double	1212	1062	IHS	②④⑥⑦⑧⑨
Gruffyd-Jones, 2001, UK	Double	1522	1299	IHS	①②③④⑥⑦⑧
Sumatriptan vs Almotriptan					
Spierings, 2001, USA	Double	1175	-	IHS	②③④⑤⑥⑦⑧⑨
Sumatriptan vs Naratriptan					
Gobel, 2000, Multinational	Double	247	127	IHS	⑥⑦⑧⑨
Zolmitriptan vs Almotriptan					
Goadsby, 2007, Italy	Double	1062	902	-	③④⑥⑦⑨
Sumatriptan vs Zolmitriptan vs Placebo					
Geraud, 2000, Multinational	Double	558	472	-	①②③④⑥⑦⑧⑨
Sumatriptan vs Almotriptan vs Placebo					
Dodick, 2002, Multinational	Double	292	249	IHS	③⑥
Dowson, 2004, UK	Double	295	-	IHS	③⑧
Sumatriptan vs Rizatriptan vs Placebo					
Goldstein, 1998, USA	Double	441	-	IHS	①②③④⑤⑦⑧⑨
Kolodny, 2004, USA	Double	1104	-	IHS	③⑧⑨
Tfelt-Hansen, 1998, Multinational	Double	548	441	IHS	①②③④⑤⑥⑦⑧⑨
Sumatriptan vs Naratriptan vs Placebo					
Dahlof, 1998, Multinational	Double	840	701	IHS	③④⑤⑥⑦⑧
Havanka, 2000, Multinational	Double	189	168	IHS	②④⑤⑥⑦⑧
Sumatriptan vs Sumatriptan-Naproxen vs Placebo					
Brandes, 2007, USA	Double	721	613	-	③④⑤⑥⑦⑨
Smith, 2005, Germany	Double	471	422	IHS	①②③④⑤⑥⑦⑨
Sumatriptan vs Eletriptan vs Placebo					
Mathew, 2003, Multinational	Double	1250	1079	IHS	①②③④⑤⑥⑦⑧
Sumatriptan vs Diclofenacpotassium vs Placebo					
DK/SMSG, 1999, Multinational	Double	220	-	IHS	⑤⑦⑧⑨
Sumatriptan vs Aspirin vs Placebo					
Diener, 2004, Multinational	Double	287	238	IHS	③④⑤⑥⑦⑧
Zolmitriptan vs Rizatriptan vs Placebo					
Pascual, 2000, Multinational	Double	909	788	IHS	①②③④⑤⑦⑧
Zolmitriptan vs Eletriptan vs Placebo					
Steiner, 2003, Multinational	Double	549	460	IHS	①②③④⑤⑦⑧
Rizatriptan vs Naratriptan vs Placebo					
Bomhof, 1999, Multinational	Double	308	262	IHS	①②③④⑤⑦⑧⑨
Rizatriptan vs Ibuprofen vs Placebo					
Misra, 2007, India	Double	103	76	IHS	③④⑥⑧
Sumatriptan vs Ibuprofen vs Aspirin vs Placebo					
Diener, 2004, Multinational	Double	287	238	IHS	③④⑤⑥⑦⑧

① 1 h pain free; ② 1 h pain relief; ③ 2 h pain free; ④ 2 h pain relief; ⑤ 2 h absence of nausea; ⑥ rescue mediaction; ⑦ recurrence; ⑧ all-adverse events; ⑨ nausea

### Statistical analysis

We initially carried out a conventional pair-wise meta-analysis which directly compares each pair of treatments. The corresponding odds ratios (ORs) and 95% confidence intervals (CIs) for each study were pooled in order to obtain the overall effect size. Furthermore, a NMA was performed for each endpoint with a Bayesian framework using R 3.2.3 software. Treatment efficacy was compared through direct and indirect evidence using the ORs and 95% credible intervals (CrIs). Then the surface under curve ranking area (SUCRA) was created to rank those interventions. The ranking probabilities were defined as cumulative probabilities with each intervention being ranked. For each endpoint, an intervention is more desirable than others with a larger SUCRA value.

## Results

### Trial eligibility

We included double-blind RCTs to investigate the treatment effects of triptans and NASIDs for adults according to the International Classification of Headache Disorders (ICHD), ICHD-II or the International Headache Society (IHS) criteria.

### Characteristic of included studies

All studies included were double-blind RCTs involving 1 four-arm trials with 287 participants and 17 three-arm trials with 9,085 participants in all. The remaining 70 studies were two-arm trials that involve 13 comparisons and a total of 34,850 participants. A detailed list of included studies, patients and diagnostic criteria characteristics is provided in Table 1. All included studies were published between 1993 and 2016.

### Pairwise comparisons

We completed pairwise meta-analysis for the 25 comparisons and the weighted ORs for each comparison were calculated. The results of the pair-wise comparisons are shown in Table 2 which illustrates the results of comparison of all 25 direct two-arm trials.

**Table 2:** Direct MA comparison of migraine treatments

Comparison	1 h-pain-free	1 h-pain-relief	2 h-pain-free	2 h-pain-relief	2 h-nausea-absence	Rescue medication	Recurrence	All-adverse event	Nausea
Sumatriptan vs Placebo	**2.89 (1.74, 4.81)**	**1.71 (1.34, 2.19)**	**2.93 (2.49, 3.44)**	**1.94 (1.76, 2.14)**	1.08 (0.90, 1.30)	**0.62 (0.54, 0.71)**	**1.34 (1.1, 1.63)**	**1.88 (1.59, 2.23)**	**1.83 (1.45, 2.32)**
Zolmitriptan vs Placebo	**2.45 (2.08, 2.90)**	**1.94 (1.73, 2.18)**	**2.75 (2.06, 3.68)**	**2.17 (1.88, 2.50)**	**1.44 (1.29, 1.62)**	**0.56 (0.51, 0.62)**	1.09 (0.67, 1.76)	**1.94 (1.49, 2.53)**	**2.17 (1.51, 3.12)**
Almotriptan vs Placebo	1.99 (0.99, 3.99)	1.32 (0.89, 1.94)	**1.66 (1.09, 2.53)**	**1.57 (1.29, 1.90)**	1.37 (0.97, 1.93)	0.53 (0.23, 1.21)	1.58 (0.87, 2.84)	1.48 (0.94, 2.33)	0.57 (0.21, 1.53)
Rizatriptan vs Placebo	**3.25 (1.79, 5.90)**	**1.71 (1.21, 2.43)**	**5.36 (4.09, 7.04)**	**2.12 (1.74, 2.58)**	**1.39 (1.24, 1.55)**	**0.60 (0.42, 0.87)**	**1.43 (1.14, 1.8)**	**1.68 (1.44, 1.96)**	1.24 (0.88, 1.76)
Naratriptan vs Placebo	3.50 (0.43, 28.8)	**1.72 (1.16, 2.56)**	**2.75 (1.66, 4.56)**	**1.99 (1.50, 2.62)**	**1.25 (1.00, 1.57)**	**0.52 (0.38, 0.71)**	1.02 (0.61, 1.7)	**1.81 (1.10, 2.98)**	1.00 (0.25, 4.08)
Ibuprofen vs Placebo	**3.14 (1.31, 7.54)**	**2.77 (1.68, 4.55)**	**2.31 (1.70, 3.14)**	**1.83 (1.32, 2.53)**	1.22 (0.97, 1.53)	**0.51 (0.35, 0.74)**	0.82 (0.4, 1.69)	1.01 (0.54, 1.87)	0.77 (0.55, 1.09)
Sumatriptan-Naproxen vs Placebo	3.37 (0.5, 22.48)	1.66 (0.8, 3.41)	**2.81 (2.11, 3.74)**	**2.21 (1.87, 2.61)**	1.09 (0.96, 1.23)	**0.48 (0.42, 0.57)**	**0.55 (0.39, 0.78)**	1.88 (0.82, 4.31)	1.12 (0.62, 2.03)
Eletriptan vs Placebo	**18.4 (4.54, 74.9)**	**3.63 (2.23, 5.92)**	**7.41 (5.16, 10.63)**	**2.70 (2.23, 3.27)**	**1.30 (1.11, 1.53)**	**0.38 (0.30, 0.48)**	**1.52 (1.16, 2)**	0.91 (0.75, 1.11)	**0.19 (0.04, 0.85)**
Diclofenacpotassium vs Placebo	2.00 (0.79, 5.05)	4.00 (0.46, 35.0)	**3.10 (2.02, 4.74)**	**3.68 (2.71, 5.01)**	1.32 (0.91, 1.91)	**1.40 (1.01, 1.94)**	**1.64 (1.25, 2.15)**	1.02 (0.60, 1.74)	0.48 (0.17, 1.41)
Aspirin vs Placebo	-	3.04 (1.87, 4.96)	**2.07 (1.57, 2.72)**	**1.56 (1.29, 1.89)**	1.07 (0.84, 1.37)	**0.66 (0.56, 0.79)**	1.09 (0.81, 1.46)	**2.31 (1.15, 4.64)**	2.99 (0.60, 15.0)
Zolmitriptan vs Sumatriptan	0.86 (0.66, 1.12)	1.01 (0.88, 1.14)	0.97 (0.82, 1.14)	1.00 (0.90, 1.11)	-	0.97 (0.68, 1.40)	1.06 (0.89, 1.26)	1.08 (0.97, 1.21)	1.11 (0.70, 1.76)
Almotriptan vs Sumatriptan	-	0.98 (0.78, 1.22)	0.79 (0.64, 0.97)	1.02 (0.85, 1.24)	1.03 (0.85, 1.25)	1.13 (0.93, 1.37)	1.16 (0.9, 1.5)	0.52 (0.37, 0.73)	0.64 (0.32, 1.30)
Rizatriptan vs Sumatriptan	**1.49 (1.16, 1.91)**	1.11 (0.95, 1.30)	**1.20 (1.08, 1.34)**	1.03 (0.91, 1.16)	1.08 (0.96, 1.23)	0.90 (0.63, 1.28)	1.07 (0.93, 1.24)	0.88 (0.75, 1.04)	0.74 (0.55, 1.00)
Naratriptan vs Sumatriptan	-	0.98 (0.63, 1.50)	0.96 (0.56, 1.62)	0.95 (0.72, 1.27)	1.02 (0.78, 1.33)	1.35 (0.79, 2.31)	0.61 (0.47, 0.79)	0.94 (0.70, 1.26)	0.78 (0.26, 2.28)
Ibuprofen vs Sumatriptan	1.87 (0.90, 3.89)	1.30 (0.87, 1.96)	0.90 (0.62, 1.30)	1.09 (0.80, 1.49)	-	1.01 (0.71, 1.43)	0.84 (0.53, 1.32)	1.07 (0.07, 17.2)	-
Sumatriptan-Naproxen vs Sumatriptan	2.03 (0.91, 4.54)	1.28 (0.86, 1.91)	**1.41 (1.16, 1.72)**	**1.20 (1.04, 1.40)**	1.07 (0.93, 1.24)	**0.66 (0.55, 0.79)**	**0.65 (0.52, 0.81)**	-	1.04 (0.59, 1.84)
Eletriptan vs Sumatriptan	1.42 (0.93, 2.15)	**1.29 (1.05, 1.58)**	**1.35 (1.10, 1.65)**	1.12 (0.96, 1.31)	1.09 (0.94, 1.27)	**0.74 (0.59, 0.93)**	0.94 (0.75, 1.18)	0.83 (0.69, 1.01)	-
Diclofenacpotassium vs Sumatriptan	1.19 (0.54, 2.63)	-	-	-	1.25 (0.87, 1.81)	-	0.88 (0.54, 1.43)	0.43 (0.26, 0.71)	0.67 (0.15, 3.03)
Aspirin vs Sumatriptan	-	1.43 (0.97, 2.13)	0.83 (0.59, 1.16)	0.97 (0.76, 1.25)	1.03 (0.71, 1.50)	1.09 (0.83, 1.42)	1.01 (0.73, 1.4)	2.55 (0.11, 61.41)	-
Almotriptan vs Zolmitriptan	-	-	0.90 (0.73, 1.11)	0.93 (0.77, 1.12)	-	0.99 (0.74, 1.32)	1.07 (0.8, 1.42)	-	1.18 (0.52, 2.65)
Rizatriptan vs Zolmitriptan	1.22 (0.73, 2.02)	1.20 (0.88, 1.63)	1.22 (0.90, 1.66)	1.05 (0.81, 1.35)	1.12 (0.87, 1.44)	-	0.96 (0.68, 1.36)	0.89 (0.63, 1.27)	-
Eletriptan vs Zolmitriptan	1.59 (0.96, 2.64)	**1.39 (1.06, 1.81)**	**1.93 (1.50, 2.49)**	1.13 (0.93, 1.38)	1.10 (0.91, 1.34)	-	0.92 (0.68, 1.23)	1.08 (0.85, 1.37)	-
Naratriptan vs Rizatriptan	**0.35 (0.14, 0.84)**	0.73 (0.49, 1.08)	**0.46 (0.31, 0.69)**	**0.70 (0.51, 0.97)**	0.86 (0.63, 1.18)	-	**0.63 (0.41, 0.96)**	0.70 (0.44, 1.09)	0.47 (0.17, 1.28)
Ibuprofen vs Rizatriptan	-	-	0.86 (0.40, 1.85)	0.72 (0.39, 1.35)	-	1.75 (0.82, 3.74)	-	0.91 (0.33, 2.53)	-
Aspirin vs Ibuprofen	-	1.10 (0.75, 1.61)	0.81 (0.54, 1.19)	0.87 (0.64, 1.19)	-	1.09 (0.77, 1.53)	1.05 (0.66, 1.68)	1.05 (0.66, 1.68)	-

Values in bold indicate significant difference

There were a total of 39,004 participants in the placebo controlled trials Direct placebo comparison results suggest all treatments are more effective than placebo with statistical significance in regards to 2 h-pain-free and 2 h-pain-relief (OR > 1, 95% CI excludes 1). All except diclofenac-potassium and almotriptan perform use of rescue medication and most drugs examined show efficacy in 1 h-pain-free and 1 h-pain-relief. Sumatriptan, zolmitriptan, rizatriptan, naratriptan and aspirin also show an increase in all-adverse events indicating some side effects.

From pairwise meta-analysis between different medications, rizatriptan is more efficacious than naratriptan concerning 1 h-pain-free, 2 h-pain-free and 2 h-pain-relief (OR < 1, 95% CI excludes 1). However, naratriptan manifests a lower recurrence than rizatriptan. Sumatriptan has a worse performance than sumatriptan-naproxen and eletriptan with respect to 2 h-pain-free and use of rescue medication. We can derive that rizatriptan and eletriptan tend to show effective performance with respect to outcomes including 1 h-pain-relief and rescue medication. However, a pairwise meta-analysis provides limited information and does not enable us to synthesize indirect evidence. Therefore we subsequently carried a NMA for further information so that all treatments could be compared and ranked.

### Network meta-analysis

**Fig. 3:**
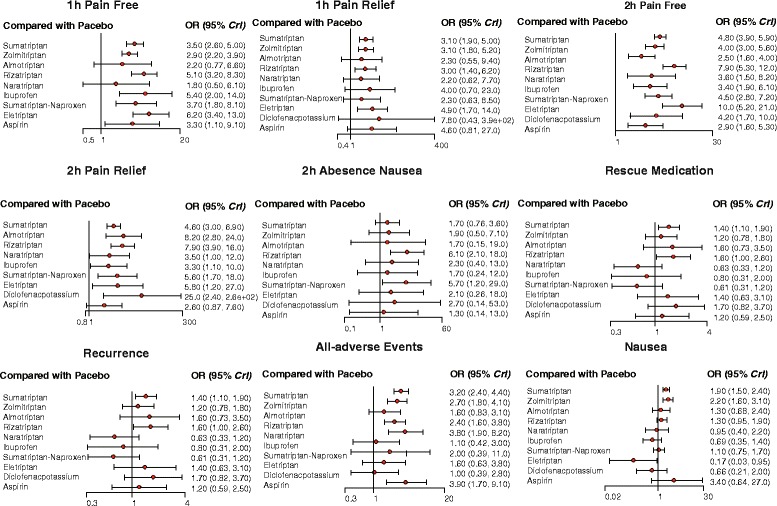
Odds ratios (95% credential intervals) for network comparison of migraine treatments

As suggested in Table 3 and Fig. 3, a large number of comparisons were generated by the NMA. As for 1 h-pain-free, all medication except almotriptan and naratriptan show statistical difference over placebo (Additional file 2: Figure S1). Furthermore, zolmitriptan appears to be less effective than rizatriptan and eletriptan, while other comparisons show no significant statistical difference. Likewise, results from NMA with respect to 1 h-pain-relief, only sumatriptan, zolmitriptan, rizatriptan and eletriptan show efficacy when compared with placebo but there were no statistical differences between any two of them.

**Table 3:** Network meta-analysis results of migraine treatments

1 h-Pain-Free	**A**	**0.32 (0.20, 0.51)**	**0.33 (0.19, 0.55)**	0.43 (0.11, 1.77)	**0.34 (0.16, 0.70)**	0.45 (0.13, 1.58)	0.25 (0.04, 1.44)	0.43 (0.12, 1.66)	**0.21 (0.07, 0.58)**	0.15 (0.00, 2.63)	0.22 (0.04, 1.28)	1 h-Pain-Relief
	**0.29 (0.21, 0.38)**	**B**	1.01 (0.53, 1.92)	1.35 (0.33, 5.59)	1.04 (0.48, 2.26)	1.39 (0.39, 5.13)	0.78 (0.13, 4.65)	1.33 (0.36, 5.22)	0.64 (0.21, 1.96)	0.46 (0.01, 8.52)	0.67 (0.11, 4.04)	
	**0.35 (0.26, 0.46)**	1.22 (0.86, 1.75)	**C**	1.34 (0.30, 5.83)	1.03 (0.43, 2.44)	1.39 (0.36, 5.28)	0.77 (0.12, 4.82)	1.32 (0.33, 5.53)	0.63 (0.21, 1.91)	0.46 (0.01, 8.13)	0.66 (0.11, 4.10)	
	0.46 (0.15, 1.23)	1.61 (0.53, 4.62)	1.32 (0.43, 3.68)	**D**	0.77 (0.16, 3.68)	1.04 (0.16, 6.61)	0.58 (0.06, 5.14)	0.99 (0.15, 6.80)	0.47 (0.08, 2.81)	0.34 (0.01, 7.95)	0.51 (0.05, 4.80)	
	**0.20 (0.12, 0.32)**	0.71 (0.46, 1.09)	**0.58 (0.35, 0.93)**	0.44 (0.15, 1.43)	**E**	1.33 (0.35, 5.08)	0.75 (0.11, 4.98)	1.28 (0.29, 5.75)	0.61 (0.17, 2.15)	0.44 (0.01, 8.67)	0.65 (0.10, 4.36)	
	0.56 (0.16, 1.93)	1.98 (0.61, 6.77)	1.61 (0.47, 5.55)	1.24 (0.25, 6.38)	2.80 (0.91, 9.23)	**F**	0.56 (0.07, 4.92)	0.97 (0.16, 5.73)	0.46 (0.09, 2.30)	0.33 (0.01, 7.03)	0.48 (0.06, 4.25)	
	**0.18 (0.07, 0.47)**	0.64 (0.25, 1.68)	0.53 (0.20, 1.41)	0.41 (0.10, 1.67)	0.91 (0.33, 2.58)	0.33 (0.07, 1.47)	**G**	1.76 (0.20, 15.48)	0.82 (0.11, 6.35)	0.57 (0.01, 18.01)	0.87 (0.11, 6.39)	
	**0.28 (0.13, 0.55)**	0.98 (0.46, 2.01)	0.81 (0.37, 1.69)	0.62 (0.17, 2.17)	1.40 (0.59, 3.12)	0.50 (0.12, 1.96)	1.51 (0.46, 4.84)	**H**	0.48 (0.09, 2.46)	0.34 (0.01, 7.70)	0.50 (0.06, 4.55)	
	**0.17 (0.08, 0.30)**	0.59 (0.30, 1.07)	**0.48 (0.25, 0.85)**	0.36 (0.10, 1.25)	0.83 (0.38, 1.71)	0.29 (0.07, 1.10)	0.91 (0.29, 2.62)	0.59 (0.23, 1.50)	**I**	0.73 (0.02, 16.01)	1.04 (0.14, 8.29)	
	**-**	**-**	**-**	**-**	**-**	**-**	**-**	**-**	**-**	**J**	1.50 (0.05, 77.84)	
	**0.31 (0.11, 0.79)**	1.07 (0.40, 2.87)	0.88 (0.31, 2.39)	0.67 (0.16, 2.95)	1.51 (0.53, 4.36)	0.55 (0.11, 2.45)	1.67 (0.58, 4.74)	1.09 (0.33, 3.76)	1.85 (0.60, 5.91)	-	**K**	
2 h-Pain-Free	**A**	**0.31 (0.25, 0.41)**	-	**0.22 (0.07, 0.72)**	**0.29 (0.17, 0.39)**	**0.27 (0.18, 0.37)**	**0.23 (0.20, 0.86)**	**0.24 (0.10, 0.30)**	**0.16 (0.12, 0.21)**	**0.03 (0.01, 0.05)**	**0.44 (0.31, 0.50)**	2 h-Pain-Relief
	**0.21 (0.17, 0.26)**	**B**	-	0.70 (0.26, 1.98)	0.89 (0.47, 1.52)	0.89 (0.44, 1.43)	0.80 (0.67, 2.08)	**0.87 (0.25, 0.99)**	**0.48 (0.39, 0.73)**	**0.08 (0.03, 0.21)**	1.42 (0.75, 2.01)	
	**0.25 (0.18, 0.34)**	1.18 (0.83, 1.68)	**C**	-	-	-	-	-	-	-	-	
	**0.39 (0.25, 0.62)**	1.90 (1.17, 3.04)	1.61 (0.95, 2.68)	**D**	1.28 (0.24, 5.80)	1.01 (0.36, 5.46)	1.52 (0.34, 4.55)	0.71 (0.41, 3.52)	0.69 (0.19, 2.78)	**0.16 (0.01, 0.39)**	1.76 (0.57, 6.84)	
	**0.13 (0.08, 0.19)**	**0.61 (0.40, 0.92)**	**0.51 (0.31, 0.84)**	**0.32 (0.18, 0.58)**	**E**	0.93 (0.68, 1.51)	0.99 (0.51, 3.17)	0.70 (0.38, 1.73)	**0.60 (0.39, 0.81)**	**0.08 (0.06, 0.17)**	**1.40 (1.14, 2.38)**	
	**0.28 (0.12, 0.65)**	1.33 (0.58, 3.17)	1.13 (0.47, 2.80)	0.70 (0.27, 1.83)	2.20 (0.93, 5.29)	**F**	0.84 (0.54, 4.69)	0.76 (0.56, 1.14)	0.52 (0.42, 1.12)	**0.10 (0.04, 0.19)**	**1.63 (1.25, 1.81)**	
	**0.30 (0.16, 0.53)**	1.42 (0.77, 2.63)	1.20 (0.61, 2.35)	0.75 (0.36, 1.58)	**2.33 (1.18, 4.69)**	1.06 (0.39, 2.88)	**G**	1.19 (0.12, 1.22)	**0.56 (0.24, 0.95)**	**0.08 (0.03, 0.25)**	1.98 (0.36, 2.40)	
	**0.22 (0.14, 0.35)**	1.06 (0.65, 1.72)	0.90 (0.51, 1.56)	0.56 (0.29, 1.07)	1.75 (0.96, 3.21)	0.80 (0.30, 2.04)	0.75 (0.36, 1.58)	**H**	0.61 (0.47, 1.98)	**0.15 (0.04, 0.26)**	**1.99 (1.38, 2.99)**	
	**0.10 (0.05, 0.19)**	**0.46 (0.23, 0.95)**	**0.39 (0.19, 0.80)**	**0.24 (0.11, 0.56)**	0.77 (0.35, 1.71)	0.35 (0.12, 1.01)	**0.33 (0.13, 0.80)**	0.44 (0.19, 1.02)	**I**	**0.13 (0.08, 0.44)**	**2.69 (1.51, 4.31)**	
	**0.23 (0.10, 0.60)**	1.12 (0.44, 2.94)	0.95 (0.37, 2.57)	0.59 (0.21, 1.67)	1.86 (0.69, 5.18)	0.84 (0.24, 2.97)	0.79 (0.27, 2.36)	1.05 (0.38, 2.99)	2.40 (0.77, 7.87)	**J**	**14.32 (9.75, 38.13)**	
	**0.35 (0.19, 0.63)**	1.67 (0.89, 3.10)	1.41 (0.72, 2.77)	0.88 (0.41, 1.88)	**2.74 (1.34, 5.69)**	1.25 (0.43, 3.43)	1.17 (0.53, 2.58)	1.56 (0.75, 3.39)	**3.58 (1.43, 9.05)**	1.48 (0.49, 4.40)	**K**	
2 h-Nausea-Absence	**A**	**0.70 (0.52, 0.92)**	0.86 (0.57, 1.29)	0.64 (0.29, 1.38)	**0.62 (0.39, 0.97)**	1.60 (0.85, 3.00)	1.25 (0.49, 3.15)	1.62 (0.84, 3.19)	0.71 (0.32, 1.61)	0.57 (0.27, 1.22)	0.83 (0.41, 1.69)	Recurrence
	0.60 (0.27, 1.29)	**B**	1.23 (0.79, 1.93)	0.92 (0.42, 1.99)	0.89 (0.54, 1.45)	**2.30 (1.22, 4.33)**	1.80 (0.71, 4.57)	**2.34 (1.20, 4.60)**	1.02 (0.44, 2.34)	0.82 (0.38, 1.79)	1.20 (0.58, 2.47)	
	0.53 (0.14, 2.00)	0.89 (0.19, 4.11)	**C**	0.75 (0.34, 1.64)	0.72 (0.40, 1.29)	1.86 (0.90, 3.85)	1.46 (0.54, 4.00)	1.90 (0.88, 4.12)	0.83 (0.35, 1.94)	0.67 (0.29, 1.57)	0.96 (0.43, 2.21)	
	0.61 (0.05, 6.60)	1.01 (0.09, 10.95)	1.14 (0.08, 18.41)	**D**	0.96 (0.40, 2.34)	2.51 (0.94, 6.76)	1.96 (0.58, 6.45)	2.54 (0.96, 6.87)	1.11 (0.38, 3.33)	0.89 (0.31, 2.65)	1.30 (0.47, 3.70)	
	**0.17 (0.05, 0.49)**	**0.27 (0.08, 0.95)**	0.31 (0.06, 1.57)	0.27 (0.02, 3.70)	**E**	**2.60 (1.25, 5.32)**	2.03 (0.73, 5.64)	**2.62 (1.19, 5.87)**	1.16 (0.46, 2.92)	0.92 (0.39, 2.23)	1.35 (0.59, 3.10)	
	0.44 (0.08, 2.48)	0.74 (0.13, 4.34)	0.84 (0.10, 7.13)	0.73 (0.04, 14.17)	2.71 (0.39, 18.41)	**F**	0.79 (0.26, 2.38)	1.01 (0.41, 2.50)	0.45 (0.16, 1.23)	**0.36 (0.13, 0.95)**	0.52 (0.21, 1.31)	
	0.58 (0.08, 4.22)	0.98 (0.12, 8.19)	1.11 (0.10, 11.19)	0.97 (0.04, 21.98)	3.57 (0.37, 35.13)	1.31 (0.10, 17.60)	**G**	1.30 (0.42, 3.94)	0.57 (0.17, 1.89)	0.46 (0.14, 1.52)	0.66 (0.23, 1.91)	
	**0.17 (0.03, 0.83)**	0.29 (0.06, 1.48)	0.33 (0.04, 2.64)	0.29 (0.02, 4.94)	1.06 (0.15, 7.12)	0.40 (0.04, 3.84)	0.30 (0.02, 3.62)	**H**	0.44 (0.15, 1.25)	**0.35 (0.13, 0.97)**	0.51 (0.20, 1.32)	
	0.47 (0.06, 3.89)	0.77 (0.09, 7.04)	0.87 (0.09, 8.93)	0.78 (0.03, 17.70)	2.82 (0.28, 29.29)	1.04 (0.07, 15.63)	0.81 (0.04, 14.26)	2.66 (0.21, 37.40)	**I**	0.80 (0.27, 2.40)	1.16 (0.40, 3.43)	
	0.37 (0.02, 7.04)	0.61 (0.03, 11.94)	0.70 (0.03, 17.74)	0.61 (0.01, 27.58)	2.22 (0.10, 54.60)	0.83 (0.03, 25.26)	0.63 (0.02, 23.22)	2.05 (0.08, 61.86)	0.79 (0.02, 28.38)	**J**	1.44 (0.52, 4.05)	
	0.73 (0.08, 6.87)	1.21 (0.12, 12.49)	1.38 (0.10, 17.99)	1.20 (0.05, 32.12)	4.40 (0.38, 54.35)	1.65 (0.10, 29.14)	1.27 (0.06, 25.07)	4.10 (0.29, 65.07)	1.58 (0.07, 35.45)	1.97 (0.05, 76.90)	**K**	
All Adverse Event	**A**	**0.53 (0.42, 0.67)**	**0.46 (0.32, 0.64)**	0.77 (0.41, 1.49)	0.75 (0.52, 1.05)	1.09 (0.45, 2.60)	1.43 (0.76, 2.86)	0.90 (0.59, 1.33)	5.81 (0.99, 35.03)	1.58 (0.54, 4.83)	0.30 (0.03, 1.52)	Nausea
	**0.31 (0.23, 0.41)**	**B**	0.86 (0.60, 1.23)	1.45 (0.77, 2.82)	1.40 (0.99, 1.97)	2.03 (0.85, 4.89)	**2.68 (1.40, 5.69)**	**1.69 (1.08, 2.60)**	**10.84 (1.88, 66.09)**	**2.96 (1.01, 9.28)**	0.56 (0.06, 2.91)	
	**0.37 (0.24, 0.54)**	1.17 (0.74, 1.87)	**C**	1.68 (0.86, 3.32)	**1.62 (1.04, 2.59)**	2.37 (0.93, 5.98)	**3.11 (1.52, 6.94)**	**1.96 (1.15, 3.31)**	**12.66 (2.11, 78.62)**	**3.44 (1.11, 11.21)**	0.64 (0.07, 3.42)	
	0.64 (0.33, 1.24)	**2.04 (1.04, 4.10)**	1.74 (0.81, 3.76)	**D**	0.96 (0.46, 1.96)	1.40 (0.47, 4.03)	1.87 (0.74, 4.87)	1.16 (0.54, 2.45)	**7.36 (1.14, 49.86)**	2.03 (0.56, 7.42)	0.39 (0.04, 2.18)	
	**0.41 (0.26, 0.63)**	1.31 (0.82, 2.10)	1.11 (0.63, 1.97)	0.64 (0.29, 1.39)	**E**	1.46 (0.60, 3.45)	1.92 (0.92, 4.30)	1.21 (0.70, 2.00)	**7.71 (1.30, 47.29)**	2.10 (0.69, 6.80)	0.39 (0.05, 2.24)	
	**0.26 (0.12, 0.54)**	0.84 (0.40, 1.72)	0.71 (0.30, 1.59)	0.41 (0.15, 1.06)	0.64 (0.29, 1.39)	**F**	1.33 (0.45, 4.04)	0.83 (0.31, 2.14)	5.36 (0.72, 40.60)	1.47 (0.35, 6.08)	0.27 (0.03, 1.85)	
	0.89 (0.33, 2.35)	**2.84 (1.06, 7.81)**	2.42 (0.86, 6.96)	1.39 (0.43, 4.47)	2.17 (0.78, 6.19)	**3.39 (1.02, 11.43)**	**G**	0.63 (0.28, 1.32)	3.99 (0.60, 26.74)	1.10 (0.30, 4.10)	0.21 (0.02, 1.16)	
	0.51 (0.10, 2.70)	1.63 (0.31, 9.07)	1.38 (0.25, 7.82)	0.79 (0.13, 4.89)	1.24 (0.22, 6.92)	1.94 (0.32, 12.28)	0.57 (0.09, 3.92)	**H**	**6.48 (1.06, 39.57)**	1.75 (0.55, 5.65)	0.34 (0.04, 1.79)	
	0.64 (0.26, 1.60)	2.05 (0.83, 5.27)	1.76 (0.68, 4.41)	1.00 (0.33, 3.08)	1.57 (0.59, 4.27)	2.45 (0.80, 7.91)	0.72 (0.19, 2.76)	1.27 (0.19, 8.49)	**I**	0.28 (0.03, 2.14)	**0.05 (0.00, 0.56)**	
	0.95 (0.35, 2.54)	**3.04 (1.12, 8.23)**	2.58 (0.90, 7.53)	1.48 (0.45, 4.84)	2.33 (0.79, 6.74)	**3.62 (1.09, 12.42)**	1.07 (0.26, 4.27)	1.85 (0.26, 12.93)	1.48 (0.39, 5.51)	**J**	**0.18 (0.02, 1.37)**	
	**0.26 (0.11, 0.58)**	0.82 (0.35, 1.91)	0.70 (0.28, 1.73)	0.40 (0.14, 1.13)	0.63 (0.25, 1.57)	0.98 (0.34, 2.92)	**0.29 (0.08, 0.97)**	0.51 (0.08, 3.19)	0.40 (0.12, 1.32)	**0.27 (0.07, 0.97)**	**K**	
Rescue Medication	**A**	0.43 (0.33, 0.56)	-	-	0.33 (0.17, 0.61)	0.52 (0.25, 1.09)	0.28 (0.14, 0.55)	0.28 (0.17, 0.46)	0.25 (0.08, 0.79)	1.63 (0.43, 6.38)	0.44 (0.24, 0.81)	
	**2.32 (1.79, 3.05)**	**B**	-	-	0.77 (0.38, 1.50)	1.21 (0.60, 2.56)	0.66 (0.31, 1.32)	0.64 (0.38, 1.10)	0.59 (0.19, 1.83)	3.81 (0.97, 15.25)	1.02 (0.54, 1.95)	
	**3.03 (1.63, 5.90)**	1.30 (0.67, 2.62)	**C**	-	-	-	-	-	-	-	-	
	**-**	-	-	**D**	**-**	**-**	**-**	**-**	**-**	**-**	**-**	
	**-**	-	-	-	**E**	1.59 (0.61, 4.19)	0.87 (0.36, 2.05)	0.84 (0.38, 1.92)	0.77 (0.21, 2.95)	5.00 (1.18, 22.48)	1.34 (0.57, 3.29)	
	1.91 (0.92, 3.97)	0.83 (0.39, 1.68)	-	-	0.63 (0.24, 1.65)	**F**	0.55 (0.20, 1.46)	0.53 (0.22, 1.27)	0.49 (0.12, 1.85)	3.15 (0.68, 14.14)	0.85 (0.32, 2.13)	
	**3.52 (1.82, 7.15)**	1.52 (0.76, 3.20)	-	-	1.15 (0.49, 2.79)	1.82 (0.69, 5.10)	**G**	0.97 (0.43, 2.31)	0.90 (0.24, 3.31)	5.77 (1.33, 27.19)	1.55 (0.67, 3.70)	
	**3.62 (2.16, 6.01)**	1.56 (0.91, 2.63)	-	-	1.20 (0.52, 2.61)	1.88 (0.79, 4.48)	1.03 (0.43, 2.34)	**H**	0.92 (0.27, 3.28)	5.92 (1.48, 25.27)	1.59 (0.72, 3.54)	
	**3.94 (1.26, 12.29)**	1.69 (0.55, 5.32)	-	-	1.30 (0.34, 4.69)	2.05 (0.54, 8.09)	1.11 (0.30, 4.22)	1.09 (0.30, 3.77)	**I**	6.50 (1.11, 37.90)	1.74 (0.48, 6.35)	
	0.61 (0.16, 2.30)	0.26 (0.07, 1.03)	-	-	**0.20 (0.04, 0.84)**	0.32 (0.07, 1.48)	**0.17 (0.04, 0.75)**	**0.17 (0.04, 0.68)**	**0.15 (0.03, 0.90)**	**J**	0.27 (0.06, 1.13)	
	**2.27 (1.23, 4.18)**	0.98 (0.51, 1.84)	-	-	0.75 (0.30, 1.76)	1.17 (0.47, 3.10)	0.64 (0.27, 1.49)	0.63 (0.28, 1.39)	0.58 (0.16, 2.07)	3.73 (0.89, 16.46)	**K**	

Treatment: A Placebo; B Sumatriptan; C Zolmitriptan; D Almotriptan; E Rizatriptan; F Naratriptan; G Ibuprofen; H Sumatriptan-Naproxen; I Eletriptan; J Diclofenacpotassium; K Aspirin

Values in bold indicate significant difference

For 2 h-pain-free, eletriptan shows efficacy over sumatriptan, zolmitriptan, almotriptan, ibuprofen and aspirin, while rizatriptan is more effective than sumatriptan, zolmitriptan, almotriptan, ibuprofen and aspirin but again there is no statistical evidence to determine the efficacy contrast between rizatriptan and eletriptan (Additional file 3: Figure S2).

Diclofenac-potassium appears to be more effective than any other intervention regarding 2 h-pain-relief. Apart from that, eletriptan also shows promising results compared with sumatriptan, rizatriptan, ibuprofen and aspirin. On the other hand, aspirin is less effective than rizatriptan, naratriptan, sumatriptan-naproxen, eletriptan and diclofenac-potassium. As a traditional treatment, aspirin is regarded as low performance in respect to 2 h-pain-relief, while diclofenac-potassium and eletriptan are outstanding treatments concerning this clinical outcome, and would be promising candidates in acute therapies.

When analyzing 2 h-nausea-absence, rizatriptan has better efficacy than sumatriptan while other treatments except Sumatriptan-Naproxen indicate no distinctive difference even compared with placebo.

Sumatriptan, diclofenac-potassium and rizatriptan present a much higher rate of recurrence figure compared with naratriptan and sumatriptan-naproxen. Furthermore, solid proof was obtained from the comparison between mono-sumatriptan and sumatriptan-naproxen that naproxen significantly reduces the migraine recurrence rate of sumatriptan while the efficacy of sumatriptan is barely influenced, and further experiments could be designed to investigate this mechanism and to combine treatments with a view to improve their preventive abilities.

Rescue medication data demonstrated that diclofenac-potassium performs the worst compared with rizatriptan, ibuprofen, sumatriptan-naproxen and eletriptan, thus diclofenac-potassium has the most likelihood of all treatments to require a rescue medication. Considering that naproxen has a notable promotion on the tolerability of sumatriptan and that diclofenac-potassium has outstanding behaviors with respect to efficacy, it is desirable to design further experiments to enhance the tolerability of diclofenac-potassium (Additional file 4: Figure S3).

Similarly, sumatriptan demonstrates a high all-adverse-event behavior compared with diclofenac-potassium, ibuprofen and almotriptan. Likewise naratriptan also has a poor all-adverse-event perform when compared with ibuprofen and diclofenac-potassium. In other words, diclofenac-potassium and ibuprofen are milder when compared with naratriptan and sumatriptan, which may indicate that NASIDs offer treatments with less adverse reactions. Aside from this, the combination of sumatriptan and naproxen appears to provide patients with much better tolerance in comparison to sumatriptan alone.

With respect to nausea, zolmitriptan and sumatriptan were significantly inferior to ibuprofen, sumatriptan-naproxen, eletriptan and diclofenac-potassium. Interestingly, eletriptan performs better than several other triptans (Additional file 5: Figure S4).

**Fig. 4:**
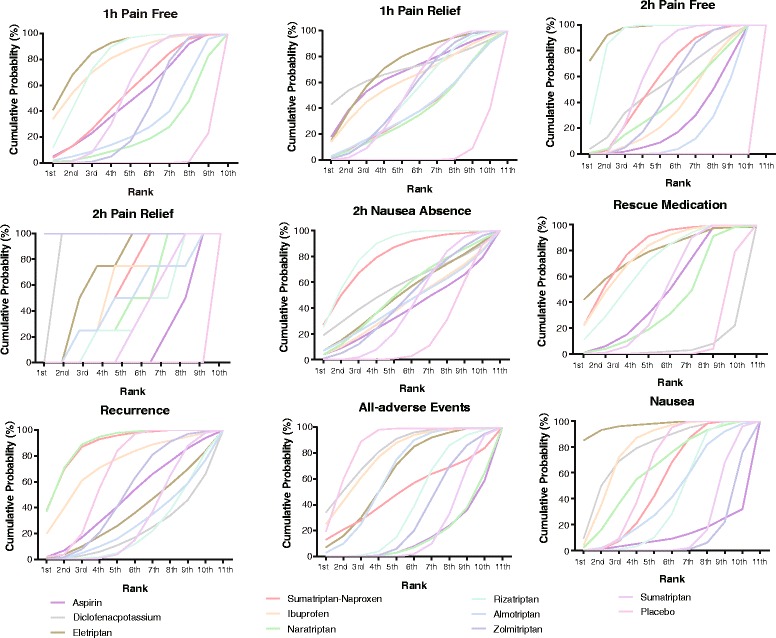
Probability of each medication with specific rank (1–11) for outcomes. Ranking indicates the probability to be the best treatment, the second best and so on. Rank 1st is best and Rank 11th is worst

Finally, Fig. 4 provides the ranking diagrams showing probability of each strategy ranked (1–11) for outcomes and Table 4 provides SUCRA results for further comparison. In general, NASIDs show a more prominent tolerability while some triptans such as rizatriptan and eletriptan exhibit more promising efficacy results. On the other hand, almotriptan has the least effectiveness with respect to 1 h-pain-free and 2 h-pain-free. Similar rankings are displayed in Table 3, which reveals that diclofenac-potassium and eletriptan has the best efficacy whereas naratriptan and almotriptan are the least efficacious medications.

**Table 4:** The SUCRA results of 11 migraine treatments on 9 clinical outcomes

Treatment	1 h-pain-free	1 h-pain-relief	2 h-pain-free	2 h-pain-relief	2 h-nausea-absence	Rescue medication	Recurrence	All-adverse event	Nausea
Placebo	0.024	0.045	0.000	0.000	0.178	0.092	0.602	0.782	0.561
Sumatriptan	0.490	0.483	0.602	0.318	0.376	0.418	0.280	0.189	0.165
Zolmitriptan	0.343	0.476	0.460	0.909	0.418	-	0.457	0.294	0.095
Almotriptan	0.282	0.358	0.186	0.432	0.394	-	0.249	0.589	0.388
Rizatriptan	0.712	0.458	0.824	0.341	0.765	0.602	0.203	0.367	0.358
Naratriptan	0.208	0.344	0.397	0.409	0.467	0.337	0.808	0.137	0.569
Ibuprofen	0.716	0.558	0.356	0.455	0.385	0.678	0.662	0.711	0.715
Sumatriptan-Naproxen	0.493	0.354	0.537	0.523	0.719	0.706	0.801	0.452	0.476
Eletriptan	0.783	0.664	0.874	0.636	0.455	0.689	0.322	0.587	0.880
Diclofenacpotassium	-	0.642	0.504	0.818	0.516	0.040	0.190	0.743	0.705
Aspirin	0.452	0.608	0.262	0.159	0.342	0.417	0.429	0.133	0.103

## Discussion

In this NMA, 10 medications were included and the result reveals that eletriptan offers the best efficacy and acceptable tolerability. Besides, our research indicates that ibuprofen exhibited the most desirable tolerability. Furthermore, diclofenac-potassium and sumatriptan-naproxen also showed favorable properties concerning efficacy and tolerability.

Triptans were a group of 5-HT_1B/1D_ agonists [102], three main mechanisms of them were all conduced to anti-migraine function. Firstly, triptans attenuated the release of vasoactive peptides trigeminal system, as well as reduced the migraine vascular inflammation. Moreover, triptans were shown to potentially inhibit the nociceptive pathway of central sensitization, thus helped to relieve the pain from migraine [103, 104].

Considering the primary efficacy end-point, triptans perform equally well compared to NSAIDs though eletriptan has the best efficacy, which lends credence to the findings of Chris Cameron et al.’s 2015 study in principle [105]. However, this study did not take adverse events into account. Therefore, we apply 4 adverse-events to characterize this ability in all 10 medications. Additionally we also included a double-component therapy. Here we report this NMA, revealing both the efficacy and the tolerability of present medication against migraine.

At first, we focused on the differences apparent in the primary efficacy end-point between different types of medications. Rizatriptan provides relatively good freedom from pain and nausea though with poor pain relief, the reason for that might be the different criteria for efficacy in each study. When it comes to tolerability, NASIDs seem to be more attractive solutions. Also, it is of significance that this study found naproxen is capable of significantly improving the tolerability of sumatriptan and has no influence on its efficacy.

As suggested by the rank probability of SUCRA, eletriptan exhibited the most considerable efficacy. From the SUCRA data, it is obvious that eletriptan can reduce pain with a better result than any other medication. In the meantime it also performs better than most of others in 1 h pain-free and 2 h pain-free. Eletriptan is a new 5-HT_1B/1D/1F_-selective receptor agonist with a higher affinity to the receptors when compared with other triptans [106]. Besides, more rapid and consistent absorption has been achieved through structural design, and this has made it possible for the drug to pass through the blood-brain barrier [107]. As a result of its enhanced hydrophobility, higher bioavailability and longer plasma half-life have also been reported [108]. When compared with sumatriptan and other triptans, the difference in efficacy may be explained by the overcoming of the blood-brain barrier, which leads to a faster and more consistent absorption [107].

When we turn attention to the NSAIDs, ibuprofen attracted us by its superior tolerability amongst all observed medications. Though ibuprofen has been available as a non-prescription medication for more than 40 years, the mechanism of how the drug works is still not completely understood. According to a widely accepted theory, it may be related to prostaglandin synthetase inhibition, therefore allowing better tolerability. From the SUCRA data we can observe that ibuprofen ranked top three in all the adverse-event indications.

The results of SUCRA also showed that the diclopenac potassium performs with high efficacy and tolerability, in fact it stood the best option among NSAIDs from a comprehensive point of view. At the mechanism level, NSAIDs inhibit the activity of cyclooxygenase (COX), which is recognized as being composed of two isoforms (COX-1& COX-2). COX acts as a catalyst during the production for prostaglandins, a substance responsible for pain and inflammation. Diclofenac inhibit both COX isoforms, though with a lower activity for COX-2 [109, 110].

Meanwhile we recognized that sumatriptan-naproxen also offered a high-level tolerability, ranking first in rescue medication and recurrence and fourth in the other two indications among the ten medications. The addition of naproxen significantly improved the tolerability of sumatriptan. To understand the reason for this we referred to the mechanism of both the medicines and the migraine.

The pathophysiology mechanism of migraine is quite complex, involving multiple neural pathways that appear to be pivotal during the process [111]. In the early stage of a migraine, vasoactive and substances including calcitonin gene-related peptide and kinins are released by trigeminal nerve endings under the stimulation of cortical spreading depression. At the same time, the central pathways’ activation depends on the signals of pain from the periphery. However, in the later stage, central sensitization has no relationship with peripheral neural input [112].

Considering the multiple pathogenic mechanisms, multi-mechanism-targeted therapy may have better effect than monotherapy. Triptans not only decrease transmission of the pain impulses to the trigeminal nucleus caudalis but also release inflammatory mediators from trigeminal nerves, therefore reduce calcitonin gene-related peptide-mediated vasodilation [113]. As for naproxen, it suppressed sensitization of central trigeminovascular neurons in the spinal trigeminal nucleus [20]. In our study, the combination of sumatriptan and naproxen effectively altered peripheral activation of central pathways during early period and development of central sensitization during later periods. As a result, a high-level tolerability was observed, and this possibility was supported by several clinical studies [114–116].

What attracted our attention most is the potentiality to combine different medications together and make use of the advantages of each considering the relevant stage. Applying different medications to intervene at every key point of the multiple pathogenic mechanisms may bring us closer to a better outcome. For instance, we may combine naproxen with eletriptan to attain treatments with both optimal efficacy and tolerability.

As with all analyses, we still cannot avoid several limitations. First, age and gender was not under consideration. Further research is needed in assessing the efficacy for different groups of people. Second, long-term assessment is also important. As we all know, people who suffer from migraine may undergo a long-period treatment and therefore the efficacy and safety of medications are vital. Third, the dosage of medications was not considered and that may cause some deviations.

## Conclusions

In conclusion, through this NMA we came to the interpretation that eletriptan may be the most suitable therapy for migraine from a comprehensive point of view. In the meantime ibuprofen may also be a good choice for its excellent tolerability. Multi-component medication also attracted our attention and it may be a promising orientation for the next generation of medication for migraine.

### Article highlights

1. Uses NMA to analysis the efficacy and tolerability of NSAIDs and triptans in migraine.
2. Eletriptan may be the most preferable treatment for migraine from a comprehensive point of view.
3. Ibuprofen has the best tolerability among all the medications.
4. Multi-component medication may be a good choice for the migraine medication in the future.

## Additional files

Additional file 1: Table S1. Jadad scale table of 88 included studies (DOCX 20 kb)
Additional file 2: Figure S1. Node splitting of direct and indirect comparisons according to type of interventions for 1 h clinical outcomes. (EPS 1868 kb)
Additional file 3: Figure S2. Node splitting of direct and indirect comparisons according to type of interventions for 2 h clinical outcomes. (EPS 2444 kb)
Additional file 4: Figure S3. Node splitting of direct and indirect comparisons according to type of interventions for rescue medication and recurrence. (EPS 1963 kb)
Additional file 5: Figure S4. Node splitting of direct and indirect comparisons according to type of interventions for all adverse events. Treatment: A Placebo; B Sumatriptan; C Zolmitriptan; D Almotriptan; E Rizatriptan; F Naratriptan; G Ibuprofen; H Sumatriptan-Naproxen; I Eletriptan; J Diclofenacpotassium; K Aspirin. (EPS 2065 kb)
