# Supplementary material for: Network meta-analysis of migraine disorder treatment by NSAIDs and triptans
Source: J Headache Pain. 2016 Dec 12;17(1):113. doi: 10.1186/s10194-016-0703-0 (PMC5153398; doi:10.1186/s10194-016-0703-0)
Supplement: Additional file 1: Table S1. — Jadad scale table of 88 included studies (DOCX 20 kb) [file 10194_2016_703_MOESM1_ESM.docx]

**Additional file 1: Table S1 Jadad scale table of 88 included studies**

| **Author, Year, Country** | **Randomization** | **Blinding** | **All-cause discontinued** |
| --- | --- | --- | --- |
| Comoglu, 2011, Turkey | 2 | 2 | 0 |
| Lipton, 2010, USA | 2 | 2 | 1 |
| Djupesland, 2010, UK | 2 | 2 | 1 |
| Mannix, 2009, USA | 2 | 2 | 0 |
| Freitag, 2008, USA | 2 | 2 | 1 |
| Wang, 2007, Taipei | 2 | 2 | 1 |
| Misra, 2007, India | 2 | 2 | 1 |
| Mathew, 2007, USA | 2 | 2 | 1 |
| Mannix, 2007, USA | 2 | 2 | 1 |
| Goadsby, 2007, Italy | 2 | 2 | 0 |
| Brandes, 2007, USA | 2 | 2 | 1 |
| Winner, 2006, USA | 2 | 2 | 1 |
| Wendt, 2006, USA | 2 | 2 | 0 |
| Tuchman, 2006, USA | 2 | 2 | 0 |
| Tfelt-Hansen, 2006, Denmark | 2 | 2 | 0 |
| Rothner, 2006, USA | 2 | 2 | 0 |
| Jelinski, 2006, USA | 2 | 2 | 0 |
| Goldstein, 2006, USA | 2 | 2 | 0 |
| Diener, 2006, Germany | 2 | 2 | 1 |
| Smith, 2005, Germany | 2 | 2 | 1 |
| Sheftell, 2005, USA | 2 | 2 | 1 |
| Loder, 2005, USA | 2 | 2 | 1 |
| Lipton, 2005, USA | 2 | 2 | 1 |
| Goldstein, 2005, USA | 2 | 2 | 0 |
| Gawel, 2005, Canada | 2 | 2 | 0 |
| Dodick, 2005, USA | 2 | 2 | 1 |
| Diener, 2005, Germany | 2 | 2 | 0 |
| Spierings, 2004, USA | 2 | 2 | 0 |
| Misra, 2004, India | 2 | 2 | 1 |
| Landy, 2004, UK | 2 | 2 | 1 |
| Kolodny, 2004, USA | 2 | 2 | 0 |
| Klapper, 2004, UK | 2 | 2 | 1 |
| Dowson, 2004, UK | 2 | 2 | 1 |
| Diener, 2004, Multinational | 2 | 2 | 0 |
| Barbanti, 2004, Multinational | 2 | 2 | 1 |
| Winner, 2003, USA | 2 | 2 | 1 |
| Steiner, 2003, Multinational | 2 | 2 | 0 |
| Mathew, 2003, Multinational | 2 | 2 | 0 |
| Charlesworth, 2003, UK | 2 | 2 | 0 |
| Sakai, 2002, Japan | 2 | 2 | 0 |
| Lewis, 2002, UK | 2 | 2 | 0 |
| Dowson, 2002, Multinational | 2 | 2 | 1 |
| Dowson, 2002, Multinational | 2 | 2 | 0 |
| Dodick, 2002, Multinational | 2 | 2 | 0 |
| Diener, 2002, Multinational | 2 | 2 | 1 |
| Spierings, 2001, USA | 2 | 2 | 1 |
| Gruffyd-Jones, 2001, UK | 2 | 2 | 1 |
| Codispoti, 2001, USA | 2 | 2 | 1 |
| Schulman, 2000, USA | 2 | 2 | 0 |
| Ryan Jr, 2000, North America | 2 | 2 | 0 |
| Pascual, 2000, Multinational | 2 | 2 | 0 |
| Pascual, 2000, Multinational | 2 | 2 | 1 |
| Lipton, 2000, USA | 2 | 2 | 0 |
| Lange, 2000, Germany | 2 | 2 | 0 |
| Kellstein, 2000, USA | 2 | 2 | 1 |
| Havanka, 2000, Multinational | 2 | 2 | 0 |
| Gobel, 2000, Multinational | 2 | 2 | 0 |
| Geraud, 2000, Multinational | 2 | 2 | 0 |
| Gallagher, 2000, USA | 2 | 2 | 0 |
| Tepper, 1999, Multinational | 2 | 2 | 0 |
| Peikert, 1999, Multinational | 2 | 2 | 0 |
| Diener, 1999, Germany | 2 | 2 | 0 |
| Ahrens, 1999, USA | 2 | 2 | 0 |
| The Diclofenac-K/Sumatriptan Migraine Study Group, 1999, Multinational | 2 | 2 | 0 |
| Bomhof, 1999, Multinational | 2 | 2 | 0 |
| Diamond, 1998, USA | 2 | 2 | 0 |
| Dahlof, 1998, Multinational | 2 | 2 | 0 |
| Dahlof, 1998, Multinational | 2 | 2 | 1 |
| Tfelt-Hansen, 1998, Multinational | 2 | 2 | 0 |
| Teall, 1998, Multinational | 2 | 2 | 0 |
| Myllyla, 1998, Finland | 2 | 2 | 0 |
| Goldstein, 1998, USA | 2 | 2 | 0 |
| Cady, 1998, USA | 2 | 2 | 0 |
| Tfelt-Hansen, 1995, Multinational | 2 | 2 | 0 |
| Pini, 1995, Italy | 2 | 2 | 0 |
| Salonen, 1994, Multinational | 2 | 2 | 0 |
| Salonen, 1994, Multinational | 2 | 2 | 0 |
| Nappi, 1994, Italy | 2 | 2 | 0 |
| Gross, 1994, UK | 2 | 2 | 0 |
| Henry, 1993, France | 2 | 2 | 0 |
| Bousser, 1993, France | 2 | 2 | 0 |
| Rao, 2016, USA | 2 | 2 | 0 |
| Winner, 2015, USA | 2 | 2 | 0 |
| Cady, 2015, USA | 2 | 2 | 0 |
| Bigal, 2015, USA | 2 | 2 | 0 |
| Silberstein, 2014, USA | 2 | 2 | 1 |
| Martin, 2014, USA | 2 | 2 | 1 |
| Fujita, 2014, Japan | 2 | 2 | 0 |

Each yes would score a single point, each no zero points; there were to be no fractional points. 1. The method of randomization was described in the paper, and that method was appropriate (1 extra point in randomization part). 2. The method of blinding was described, and it was appropriate (1 extra point in blinding part).
